# A Double Hit CD10-Negative B-Cell Lymphoma with t(3;8)(q27;q24) Leading to Juxtaposition of the *BCL6* and *MYC* Loci Associated with Good Clinical Outcome

**DOI:** 10.1155/2014/120714

**Published:** 2014-10-01

**Authors:** Lucinda Sanders, Sandrine Jayne, Ben Kennedy, Fiona Miall, Sietse M. Aukema, Reiner Siebert, Simon D. Wagner, Martin J. S. Dyer

**Affiliations:** ^1^Department of Haematology, University Hospitals of Leicester, Leicester LE1 5WW, UK; ^2^Ernest and Helen Scott Haematological Research Institute and Department of Biochemistry, University of Leicester, Leicester LE1 9HN, UK; ^3^Christian-Albrechts-University Kiel and Universitatsklinikum Schleswig-Holstein, Campus Kiel, Arnold Heller Straße 3, 24105 Kiel, Germany; ^4^Ernest and Helen Scott Haematological Research Institute and Department of Cancer Studies and Molecular Medicine, University of Leicester, Leicester LE1 9HN, UK

## Abstract

The WHO classification of lymphomas allows for a group of diseases that have features intermediate between those of Burkitt lymphoma and diffuse large B-cell lymphoma. These are a diverse group of diseases whose genetics and clinical course are yet to be fully described. We report an unusual case of high grade B-cell lymphoma, intermediate between DLBCL and BL, lacking CD10 expression in which the chromosomal translocation t(3;8)(q27;q24) was found to be the sole chromosomal abnormality. FISH analysis demonstrated juxtaposition of the *BCL6* and *MYC* loci without obvious involvement of the *IGH* locus, suggesting constitutive *MYC* expression due to promoter substitution. The patient responded to intensive chemotherapy and remains in remission two years after finishing therapy.

## 1. Introduction

Making a correct diagnosis of high grade B-cell lymphoma is critically important because Burkitt lymphoma (BL) is highly curable with intensive chemotherapy treatment regimens [1, 2] but not with the standard regimens employed for diffuse large B-cell lymphoma (DLBCL). BL can occur in various clinical settings (endemic/sporadic and as a consequence of immunodeficiency) and can present with either nodal or extranodal disease. Morphologically, BL (L3) blasts characteristically have deeply basophilic, vacuolated cytoplasm; typically histological sections show a “starry sky” appearance, due to tingible body macrophages [3]. Immunophenotypically, virtually all BL blasts express Ki67, indicative of a high proliferation rate, and lack BCL2 expression, possibly reflecting their origin from germinal centre B-cells. Only rarely do cases fail to express the germinal centre surface marker, CD10 [4]. Most cases coexpress TP53 protein, arising due to a variety of mechanisms, not only* TP53* mutation [5]. Cytogenetically, BL are characterized by a “simple” karyotype and specifically with chromosomal translocations involving the* MYC* locus on chromosome 8q24 with either the* IGH* locus on chromosome 14q32 or the* IGK* or* IGL* loci on chromosomes 2p12 and 22q11, respectively [6–8]. DLBCL has distinctive morphological features but is heterogeneous with respect to immunohistochemistry and gene expression [9, 10]. Histological separation of DLBCL and BL can be difficult although gene expression profiling permits the accurate classification of these two diseases [6]. However, gene expression profiling is not yet widely used for this purpose and the principle means of diagnosis is immunohistochemistry combined with FISH interphase cytogenetics.

FISH cytogenetics has revealed translocations involving the* MYC* locus which are sometimes accompanied by recurrent breaks at other loci, particularly the* BCL2* gene on chromosome 18 or the* BCL6* gene on chromosome 3.* MYC*+/*BCL2*+ cases account for 62% of double hit lymphomas, whereas* MYC*+/*BCL6*+ cases are much more rare at 8%. Morphologically double hit lymphomas can have the appearance of BL, DLBCL, or follicular lymphoma whilst immunohistochemical studies suggest that the majority coexpress CD10, BCL6, and BCL2 and have a proliferation index between 50 and 100% as assessed by Ki67 staining [11]. Double hit lymphomas tend to have high LDH and extranodal involvement at presentation and a poor response to treatment. These considerations caused the WHO to introduce a novel category of B-cell lymphoma unclassifiable with features intermediate between BL and DLBCL. Double hit lymphomas involving the BCL6 locus have been reported to be particularly aggressive [8, 12].

There are many uncertainties with respect to diagnosis and management of double hit lymphomas. Here we present a case of double hit lymphoma with unusual immunophenotypic and cytogenetic features with a good response to intensive chemotherapy.

## 2. Methods

Interphase fluorescence* in situ* hybridization (FISH) was performed as previously described [13–15]. Probes were employed as indicated in the text.

## 3. Case

A previously well 37-year-old female of southern Asian origin presented in July 2011 with a one-month history of malaise, intermittent fever, weight loss of over 5 kg, and back pain, which was sufficiently severe for initial referral to the orthopedic surgeons. On examination she was tender over the L4/5 vertebrae but with no neurological deficit. There was no palpable lymphadenopathy or hepatosplenomegaly. An MRI scan of the thoracolumbar spine showed increased signal consistent with diffuse marrow replacement. Diagnostic blood tests showed hemoglobin of 112, white cell count of 8.3 × 10^6^/L (differential: neutrophils 6.03; lymphocytes, 1.7), and platelets of 196 × 10^9^/L. Renal and liver function tests were normal but lactate dehydrogenase was markedly raised at 2029 (upper limit of normal 255). HIV and hepatitis screens were negative. Serum immunoglobulins were within the normal range and there was no detectable paraprotein. CT scan of thorax, abdomen, and pelvis was normal.

**Figure 1:**
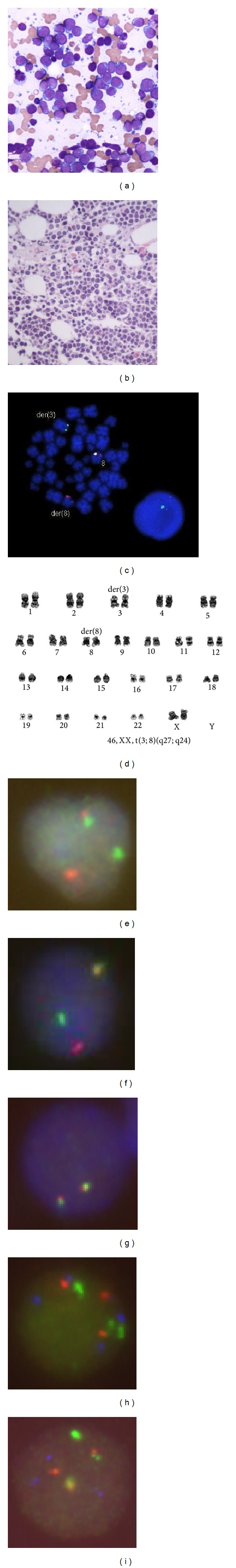
Morphology, karyotype, and FISH analysis of the case. (a) Blast cells demonstrating basophilic cytoplasm and high nuclear cytoplasm ratio. (b) Bone marrow morphology showing diffuse infiltration. (c) Analysis of bone marrow metaphase using FISH probes. A Vysis LSI* MYC* dual colour break-apart rearrangement probe was employed to show a* MYC* fusion on the normal chromosome 8, with the other MYC allele split between the der(3) and der(8). (d) G-banding karyogram showing t(3;8)(q27;q24). ((e), (f), and (g)) Interphase FISH analyses (false colour display derived from the ISIS/MetaSystems FISH system). (e) FISH analysis of the t(3;8) translocation. (e)* MYC* break-apart probe showing one red/green colocalised signal from the unrearranged locus and two separate red and green signals from the rearranged locus. (f)* BCL*6 break-apart probe showing one red/green colocalised signal from the unrearranged locus and two separate red and green signals. (g)* IGH* break-apart probe showing a signal of two colocalised red/green signals indicating no breaks at the* IGH* locus. (h)* MYC*/*IGH*/*CEP* 8 tricolor dual-fusion probe.* IGH* (green) does not colocalise with* MYC* (red). The 8p11.1-q11.1 CEP8 alpha satellite probe gives a blue signal. (i) Colocalisation of* BCL*6 (green) and* MYC* (red) without fusion of* IGH* signals (blue).

Bone marrow aspirate and trephine showed complete replacement of the normal bone marrow by blasts with L3 morphology, with basophilic cytoplasm and vacuolation (Figures 1(a) and 1(b)). Analysis of the bone marrow blasts by both flow cytometry and immunohistochemistry showed a composite immunophenotype of strong expression of CD19, CD20, and CD79A but without detectable CD10. Ki67 was seen in all cells indicative of a proliferation rate of 100%. Immunohistochemistry showed that TdT was absent as was BCL2 protein, whereas TP53 protein was detected in all cells.

Metaphase cytogenetics showed a single chromosomal translocation involving the long arms of chromosomes 3 and 8 in seven of ten metaphases; the final karyotype was 46,XX,t(3;8)(q27;q24)[7]/46,XX[3] (Figures 1(c) and 1(d)). Specific fluorescent* in situ* hybridisation (FISH) investigations were also undertaken on this sample: the Vysis LSI* MYC* dual colour break-apart rearrangement probe, which detects the majority of* MYC* breakpoints at 8q24, showed rearrangement in 39 of 100 (39%) interphase nuclei examined (Figure 1(c)).

Interphase molecular cytogenetic analysis with* BCL6*,* MYC,* and* IGH* break-apart probes (Figures 1(e), 1(f), and 1(g)) showed a signal constellation indicating a break at the* BCL6* and* MYC* loci in 12 and 11% of interphase cells, respectively, but two nonrearranged* IGH* alleles (Figures 1(e), 1(f), and 1(g)). Hybridization with a* IGH*-*MYC-CEP8* tricolor dual-fusion probe which detects the typical t(8;14)(q24;q32) showed no evidence of t(8;14)(q24;q32) (Figure 1(h)). Importantly, hybridization with probes spanning the* MYC* (red),* IGH* (blue), and* BCL6* (green) loci probes showed* MYC*-*BCL6* fusion without any detectable colocalisation or insertion of* IGH* signals in 14% of examined cells (Figure 1(i)) [13].

The patient was treated initially with combination chemotherapy according to the R-CHOP protocol, but when the additional FISH data became available this was followed by two cycles of R-CODOXM-IVAC as used in the UK LY10 study [2]. The latter chemotherapy was poorly tolerated and required several prolonged in-patient admissions with severe neutropenic sepsis and mucositis, but a posttreatment bone marrow in December 2011 demonstrated complete remission. She continues well off all specific therapy 33 months later.

## 4. Discussion

The FISH studies performed on this case showed that the t(3;8)(q27;q24) translocation resulted from the juxtaposition of upstream (telomeric)* BCL6* sequences on chromosome 3q27 with* MYC* on chromosome 8q24. This case is, therefore, best classified as B-cell lymphoma with features intermediate between DLBCL and BL [16] or highly proliferative “regular” DLBCL. The present case demonstrates that Burkitt lymphoma morphology can be associated with* MYC* translocations that do not involve the immunoglobulin loci. We suggest that the t(3;8)(q27;q24) resulted in promoter substitution with sequences on chromosome 3q27 driving deregulated* MYC* expression. This appears to have occurred as a single event without prior generation of a t(8;14)(q24;q32) translocation, although without the benefit of DNA sequence analysis we cannot exclude a small amount of* IGH* sequences inserted at the breakpoint junction.

Similar chromosomal translocations involving* BCL6* and* MYC *have been described previously, but usually in the context of more complex karyotypic abnormalities and usually in other subtypes of B-cell malignancy. To our knowledge, the first cytogenetic description of t(3;8)(q27;q24) was in an 11-year-old girl with Burkitt lymphoma/leukemia arising on a background of ataxia-telangiectasia [17]; in this case, the* BCL6/MYC* “fusion” was subsequently translocated to other chromosome arms including 1q, 6q, 12p, 18q, and Xp as the malignancy evolved. Subsequent cytogenetic and molecular descriptions of t(3;8)(q27;q24) have been in DLBCL cases with more complex karyotypes including t(14;18)(q32;q23) “triple hit” cases [18–22]; DNA sequence analysis has shown direct juxtaposition of sequences from chromosome 3q27 with those from chromosome 8q24 with no intervening* IG* sequences; the* BCL6* breakpoints fell outside the* BCL6 *major translocation cluster (MTC). It is likely that the consequences of the translocation included deregulated expression of both BCL6 and MYC. Recent reports suggest that* MYC-BCL6 *rearrangements occurring in DLBCL are associated with an aggressive clinical course [8, 12] although similar results have not been obtained in all series [23].

Germinal centre derived DLBCL often express CD10, whereas activated B-cell type does not [24, 25]. Recent reports show that double hit lymphomas involving the* BCL6* locus often lack CD10 expression but are GCB-like by immunohistochemistry according the Hans' algorithm [12, 26], although ABC-like cases have been reported by gene expression profiling [8].

In summary, we present a case of CD10 negative but otherwise typical BL with an unusual* MYC* translocation involving* BCL6* as the sole cytogenetic abnormality; treatment with BL-type therapy has resulted in a durable remission.
